# Bibliographical

**Published:** 1860

**Authors:** 


### Bibliographical.

We have received from Messrs. Blanchard & Lea, through Messrs. J. J. Richards & Co., of this city,

"*An Introduction to Practical Pharmacy,*" designed as a Text Book for the Student, and as a guide for the Physician and Pharmaceutist, with many formulas and prescriptions, by Edward Parish, graduate in Pharmacy—member of the Philadelphia College of Pharmacy and of the American Pharmaceutical Association, and principal of the School of Practical Pharmacy of Philadelphia. This is the second edition, greatly enlarged and improved, of a work, the success of the first edition of which abundantly proved its adaptation to the wants of those for whom it was written, and in its revision, it has been made more worthy of general acceptance. To keep pace with the progress of Pharmacy within the past few years, has required the most persevering industry and zeal. Since the first publication of this work, numerous chemical physiological and therapeutical discoveries have added to the resources of the physician, with a corresponding enlargement of the domain of pharmacy, while the progress of scientific education and enterprise among pharmaceutists has multiplied the numbers, and perfected the details of their processes.

Besides the increased size of the page, and the abundant use of small type in the least important parts, it has been found necessary to add nearly 200 pages of new matter, and to provide a number of original drawings in addition to the very extensive series of illustrations contained in the first edition. In these additions, the elementary character of the work, which is appropriately styled "An Introduction to Practical Pharmacy," has been kept steadily in view, and the more strictly scientific facts have been distinctly separated from those which are elementary and practical.

In developing the important relations of Pharmacy to organic Chemistry, many recent foreign works have been consulted, not generally accessible to students in this country, and it is confidently believed that the present issue will be found a fair exponent of the state of pharmaceutical science up to the time of its issue, and is commended to the favorable consideration of physicians and pharmaceutists.

*Pathological and Practical Observations on Diseases of the Alimentary Canal, Esaphagus, Stomach, Cecerne, and Intestines.* By S. O. Habershon, M. D., Fellow of the Royal College of Physicians, Assistant Physician to Guy's Hostal, &c., &c.

Diseases of the stomach have, during the last few years, received considerable attention, and our medical literature has been enriched by the labors of Budd, Handfield Jones, Chambers, Brinton and others. Much, however, still remain to be done; and whilst some of the facts contained in the present volume will tend to confirm what is already known, other *new ones* will be found, which will repay an attentive perusal of its pages. It has been designed to illustrate the diseases treated upon, by cases which came under the personal observation of the author during his extensive experience as Demonstrator of Morbid Anatomy, and Curator of the Museum at Guy's Hospital, and embraces chapters on disease of the Esophagus, Organic Diseases of the Stomach, Functional Disease of the Stomach, Diseases of the Duodenum, Muco-Enteritis and Enteritis, Strumous Disease of the Alimentary Canal, Diseases of the Cœcum and Appendix Cœci, Diarrhœa, Colitis and Dysentery, Typhoid disease of Intestine, Colic, Constipation, Internal Strangulation, Intussusception, Carcinoma of the Intestine, Intestinal worms, Perforation of Intestine from without, Abscess in the Abdominal Parietes, extending into the intestine and Fecal abscess. The contents are thus seen to embrace a most interesting series of pathological observations; and in addition to the details of the peculiarities of each case, there will be found embodied, the more general deductions and apparent conclusions derived from the whole. This work has also been received through Messrs. J. J. Richards & Co., from the publishers, Messrs. Blanchard & Lea, Philadelphia.

*The Use and Abuse of Tobacco;* By JOHN LIZARS, late Professor of Surgery to the Royal College of Surgeons, and lately Senior Operating Surgeon to the Royal Infirmary of Edinburgh. From the Eighth Edinburgh Edition.

Those, we presume, who are hopelessly abandoned to the use of the noxious weed, whose destructive effects are exhibited in this work, have been disposed to criticise it somewhat severely, but we cannot do otherwise than commend any effort which has the slightest tendency to arrest the progress of this unfortunate habit. The subjoined extract was originally published in the London Lancet, and will be found to contain a striking presentation of melancholy facts, connected with this subject, which unfortunately belong equally to its use in America, as in Europe. It is taken from a communication of Mr. Solly, addressed to the Journal alluded to, and re-published in Mr. Lizar's work.

"The more I think of the tobacco question, the more it haunts me. I feel that I cannot do justice to its importance, but I am anxious to add something to my last communication. Every day the subject is forced upon my mind. I scarcely meet a friend or patient who does not bear his testimony to the mischief of which he has been the witness, in his own case or that of some friend, from tobacco.

"The profession have no idea of the ignorance of the public regarding the nature of tobacco. Even intelligent, well-educated men, stare in astonishment, when you tell them that tobacco is one of the most powerful poisons we possess. Now, is this right? Has the medical profession done its duty? Ought we not, as a body, to have told the public that, of all our poisons, it is the most insidious, uncertain, and, in full doses, the most deadly. Why should they not know at once how often it has proved fatal in the human subject, when injected into the rectum in strangulated hernia. I heard, only the other day, that a celebrated surgeon—rather an obstinate one—since dead, lost five cases in succession from the effect of tobacco injected into the bowels.

"It seems almost trifling with the subject, and yet the extreme ignorance which prevails regarding this frightful pest, rendering even trifles weighty in the scale, induces me to remind all smokers, and those of our brethren who madly encourage it, that the first effect of a cigar on any one, demonstrates that tobacco can poison by its smoke, and through the lungs, just as certainly as through the bowels.

"It is true, that the all-perfect laws of Nature point out to careless man, that he is taking in a poison, and by the sickness, headache, and vomiting, which follow, stop for the time the poisonous dose, and avert the fatal end.

"Look at the pale face, imperfect development, and deficient muscnlar power of the inhabitants of unhealthy malarious districts. They live on, but with only half the proper attributes of life. So it is with the habitual smoker: his system is accustomed to the poison; and so the opium-eater can take an ounce of laudanum for his morning's dram, and feel it not, when the eighth part of it would be fatal to the uninitiated.

"What a blessing it would have been to mankind, if all men had shrunk from this plague of the brain, as did the first Napoleon. One inhalation was enough. In disgust he exclaimed, 'Oh, the swine! My stomach turns. It is a habit only fit to amuse sluggards.'

"It is not, however, to be denied, that when the first poisonous effect has passed of, and the system begins to tolerate it, that tobacco acts as a slight stimulant to many organs.—First to the brain, like wine and spirits in small quantities, or inflammation in its very earliest and very transitory stage, it excites to an unnatural degree the natural function of the part. I once knew a young clergyman, who could only write his sermons uuder the stimulus of tobacco, and there is no question that these discourses were brilliant, eloquent, and most interesting to listen to; but the end of that man is not yet come.

"In the same way, tobacco is a stimulus to the generative sytem; but the stimulating effect is much earlier followed by its depressing action; consequently it has long been known, when used immoderately, to extinguish the sexual appetite, and annihilate the reproductive faculty. It is a prolific source of spermatorrhœa. During one week lately, I was consulted by three young men suffering from seminal weakness, in all of whom I could trace this drain to the relaxing, enervating effect of smoking. Happy would it be for them if the abandonment of the vice would at once restore them to health; but no! the evil remains, though the cause is removed—I do not mean remains permanently, because all such cases are ultimately, though sometimes slowly, curable. These three cases are merely a few out of many I have seen of late years.

"I have been asked to produce *facts* in proof of the deleterious effects of tobacco, and facts in abundance shall be forthcoming when I have had a record kept of its effects in my hospital cases; but the facts which I have now by me being private cases, contain details the relation of which would involve a breach of confidence which nothing would justify. No man likes to be held up as a victim of tobacco smoke, though I could name many whose health has been decidedly injured by it. I have seen many cases of amaurosis, both in the incipient and advanced stage, caused by smoking.

"I know a valued servant, in a family where I attend, whose memory was failing him, his face getting yellow, and his hand shaking; so that those who did not know him, attributed his condition to drinking. He abandoned smoking, and in two years was an altered man.

"For above ten years I smoked occasionally; and I am well acquainted with all the soothing, calming, and, for the time, agreeable effect of a cigar, or even short pipe. I left it entirely off about nine years since. This I did, because I believed it impaired my nervous energy; and I have every reason to be satisfied with the change. Since that time my attention has been uninterruptedly directed to the question —Is tobacco smoking positively injurious? The conclusion, therefore, which I have briefly given to the world through your pages, has not been hastily or capriciously formed on a few isolated facts. For the last twenty years I have been the medical examiner of various insurance offices—the Royal Exchange, the Victoria, the Crown, and New Equitable. The two former I still hold. In my examinations, I inquire whether the examinees are in the habit of smoking; and I can now generally tell by the countenance whether they are or not habitual smokers. If I have any doubt on this point, an examination of the fauces decides it. The fauces of the smoker are always more or less injected and rough, presenting the appearance of a piece of dirty red velvet, instead of the pale, pinkish lilac hue of a healthy throat. The tongue, when smoking is not combined with drinking spirits, as is seldom the case in the upper and middle classes, is usually furred and white, but not otherwise unhealthy.* This con-

* The author has had a representation made, illustrating these effects.

dition of the fauces may be produced by, and always accompanies the intemperate use of, intoxicating liquors; but then the tongue is unnaturally red; the papillæ at the tip and gustatory papillæ prominent and angry. The condition of the fauces is well worthy the attention of the profession; let them notice it, if possible, in almost every patient that comes before them, and they will soon be struck with the correct index these parts afford of the habits of their possessors.—There is one source of fallacy which must, however, be guarded against. This is a temporary vascular injection, induced by the long-continned straining of some people, when requested to take a deep breath for the purpose of showing the fauces. Where, however, the examiner is aware of this fact, he will find no difficulty in distinguishing the temporary blush from the permanent stain. I may here add, by-the-by, that I have occasionally detected habits of intemperance, which the statement of the examinee, and the letters of his referees, gave no note of. In truth, there are many men who habitually drink more than is consistent with longevity, but who never get drunk. Such men invariably declare that they are quite temperate. This condition of the tongue and fauces is not limited to the mouth; it is not a mere local congestion; it exists, more or less, in the stomach, and the rest of the alimentary canal; and hence, I believe, in the otherwise healthy subject, a cigar acts as a moderate purgative, but in typhus as a poison. Can, however, any medical man assert, that it is natural or healthy to take an aperient daily? In the habitual smoker the heart is irritable, and the person nervous; the pulse frequently intermittent, and irregular in force and frequency.

"In the course of my practice, I have met many individuals, who, like myself, have abandoned smoking, because they thought it did not agree with them. Many have done so at my suggestion. I have never found one who does not assert, most positively, that he has been in better health since, and that his intellectual activity has been increased.

"With regard to the arguments that have been adduced in favor of its innocence, I will first advert to the Turks.—The mental condition of the Turks, as a nation, would be one of the strongest arguments on my side, were the question not complicated with opium. The fact of their longevity as a race must be proved by statistics, to establish the opinion that smoking does not shorten their lives; but even then it would not prove that smoking is innocuous to Englishmen.

My assertion, that it is especially injurious in England, applies to the young men of this country, about whom I am most anxious, because they all live up to fever-point. I believe that the injury inflicted by a pipe of tobacco in the mouth of a poor man, who lives below par rather than above it, cannot be appreciated; but not so a cigar smoked by a man who lives high, and uses his brain much. It matters little whether the mere animal, let him be in the shape of a stock-broker's clerk or a country voluptuary, smokes more or less; but I am sure it is incompatible with great and long-continued intellectual activity, and that amount of high living which appears almost necessary to health in the imperfect atmosphere of great towns.

"The different mode of living on the Continent and here, renders all arguments drawn from the effect of smoking on foreigners, in favor of the habit, scarcely applicable to the inhabitants of this island; though even in Holland, according to the statement of that interesting writer, Dr. Carlyon, this habit is fatal. It appears to me, that it is our *duty* to discourage any habit that is not conducive to health, and equally *criminal* to encourage a habit which is liable to become a master and a tyrant.

"The gentry and aristocracy of this country must not suppose that because the habit of smoking does not lead in their case, to drinking, that, therefore, it injures them not. Hundreds of gentlemen smoke, without drinking more than they believe is conducive to health, and smoking does not in their persons lead to intemperance. But from this fact the habit is the more dangerously insidious. Its ill effects are less easily observed; the habit advances in intensity without their perceiving any objection to it; but the penalty is paid, nevertheless, and an untimely grave is often the result.

"One of the best riders to hounds in England, who never smokes, told me that he required much less sleep than his friends, almost all of whom smoke; and that they often remarked with astonishment, how fresh he always was in the morning, notwithstanding late hours, champagne, &c. That gallant soldier, General Markham, whose life was sacrificed to his hasty journey from India, never smoked himself, nor would he allow any of his personal staff to do so, so strong was his opinion of its injurious tendency to the soldier's character.

"I may be mistaken, but I believe that all our greatest men—I mean intellectually—statesmen, lawyers, warriors, physicians, and surgeons, have either not been smokers, or if smokers, that they have died prematurely.

"My friend, Mr. Whitfield, the resident medical officer at St. Thomas' Hospital, speaks most strongly of the injury he has witnessed from habitual smoking, his experience extending over above forty years, in a hospital containing over 500 beds, and relieving some thousands of out-patients every year. He has seen three cases of delirium tremens induced by tobacco smoke alone. In none of these cases had the patients indulged in drinking intoxicating liquors, so that there was no doubt of the single cause of the disease."

We regard this work as an exceedingly interesting and valuable one, and would be pleased to know that it was in general circulation, not only among the profession, but among the people generally. It can be obtained of Messrs. Lindsay & Blakiston, Publishers, Philadelphia.

We have also to acknowledge from the same source with the above—

"*Alcohol; its Place and Power.*" By JAS. MILLER, Professor of Surgery in the University of Edinburgh—Surgeon in Ordinary to the Queen for Scotland, &c., &c.

This is taken from the Nineteenth Glasgow Edition of a work of high authority on the medical view of the "Place and Power" of Alcohol. The work was written in response to an application to Prof. Miller, by the Scottish Temperance League, to whom the learned author presented the M. S., only stipulating that it should be published at such a price as would bring it within the reach of all classes. It is a small work of most decided merit, and is readily transmissable by mail.

We also acknowledge, with great pleasure,

"*An Epitome of Braithwaite's Retrospect of Practical Medicine and Surgery*"*; in Five Parts.*

PART 1.—Containing a condensed summary of the most important cases, their treatment, and all the remedies and other useful matters embraced in the *forty volumes*—the whole being alphabetically arranged and classified, and supplied with an "Addenda," comprising a table of French weights and measures, reduced to English Standard. A list of incompatibles—explanation of the principal abbreviations occurring in Pharmaceutical formula. A vocabulary of Latin words most frequently used in prescriptions, and a copious index. The work is Edited by Walter S. Wells, M. D., and published for the author, *exclusively by subscription*, by Charles T. Evans, 114 Fulton St., N. Y.

We regard this as one of the most valuable contributions of the day, and as a special favour conferred upon the profession. Physicians desirous of subscribing to this work, can receive the first part at once (which will be sent by mail, postage paid), on their remitting $1.00, addressed to Charles T. Evans, Box 4553, New York P. O. The second and concluding parts will be issued monthly, and every subscricer will be notified when each "PART" is ready.

We have also been furnished with the following notice by an intelligent medical friend, to whom we had turned over the book alluded to:—

*A Practical Treatise on the Diagnosis, Pathology, and Treatment of Diseases of the Heart;* By AUSTIN FLINT, M. D., Prof. of Clinical Medicine, etc., etc.; Philadelphia, Blanchard & Lea, 1859.

The name of the author of the work which bears the above title, is already familiar to every medical man in the United States, and is by no means unknown in the medical circles of the British Isles and Europe, and his industry, energy and perseverance, in the investigation of Diseases of the Thoracic Viscera, warrant us in expecting a work of much interest and information. The present is the most elaborate Treatise on Heart Diseases, which has appeared in this country within the last twenty years; and certainly there is a great want for a work exclusively devoted to this most interesting, important, and hitherto little known class of Disease. The author has treated of these morbid conditions in a masterly manner and lucid style, and has thrown around these obscure diseases a charm which is seldom found in a work of the kind. The attractive manner in which the author has treated of the symptoms and physical signs of the various lesions of the Heart and its appendages, is surpassed only by the clearness of expression of his views of their Pathology, and the manner of their treatment. As an instance of the way in which Dr. Flint manages the subjects of which he treats, we instance the following extract from page 101. After having defined fatty growth and Degeneration, and given the symptoms and physical signs, he proceeds to the treatment by stating the indications, thus: "The general objects of Medical treatment in cases of fatty growth and degenera tion, are three-fold, viz: 1st, To obviate and relieve the immediate effects of weakness of the Heart. 2nd, To increase permanently the muscular power of the organ; 3rd, To arrest or limit the accumulation of fat." Then comes the treatment in such an interesting style, that no one who ever fixes the indications in his mind, can mistake or fail to remember it at the time when it is mostly needed. The book is an admirable assistant to the general auscultator of chest diseases, as well as highly valuable to him who is called to attend diseases of the Heart. The only thing we regret about the whole book is, that not more has been said with regard to the connection between diseases which affect other organs in common with the heart, or which may terminate in a lesion of that organ.

Altogether the book is the most valuable Treatise which we have, on the diseases of which it treats, and the only one which is up to the present standard of the knowledge of these diseases, as developed by the progress of science. A companion volume to Dr. Flint's late work on the respiratory organs; it is fully equal to it, and we cordially commend it to the profession. A. G. T.

We had hoped to have had a "Review," from competent hands, of Gross' Surgery, for the present number of the Journal, but have been disappointed. It will, however, appear in the March number.

